# State of the Urine during Pregnancy and Disease

**Published:** 1842-04

**Authors:** 


*State of the Urine during Pregnancy and Disease.*—M. Donne read a paper to the Academy of Sciences at their meeting of the 31st May last, the object of which was to show that the urine of pregnant women generally furnishes certain characters by which the existence of pregnancy may be ascertained. From a great number of experiments, M. Donné has ascertained that the urine contains much less free acid, phosphate and sulphate of lime, in pregnant than in other women. This circumstance modifies in a very remarkable manner the microscopic crystals of the salts contained in the urine, and enables us to determine with great probability, if not absolute certainty, the existence of pregnancy. M. Donné has applied the method successfully in more than thirty cases, at different periods of utero-gestation.

The author has also examined the properties of the urine in various diseases. In chlorosis the results obtained have been positive and very striking. Healthy urine, as we all know, contains a certain quantity of iron. In chlorosis all trace of the metal disappears; but as soon as we begin to administer the preparations of iron, it is again found in the urine. According to M. Donné, we cannot consider the cure of chlorosis complete, unless the urine contains its regular proportion of iron, some time after we have ceased to administer the remedy. In certain affections which bear some resemblance to chlorosis, but should be distinguished from it, the author found that the urine contained a considerable quantity of iron.

In cases of pulmonary consumption, a very curious fact was noticed; when the urine is evaporated, instead of leaving the ordinary crystals, it gives rise to a thick, viscid matter, analogous to that extracted from diabetic urine. The author asks whether this be saccharine matter, or some animal substance, and confesses his ignorance on the point; the fact, however is worthy of attention, especially since the observations of M. Rayer have established some analogy between diabetes and pulmonary consumption, by proving that the latter generally ensues on the apparent cure of the former. However, this appearance of the urine is so constant and remarkable that it will often enable us to diagnosticate the existence of phthisis, even without seeing the patient.

The microscopic crystals of the urine in cases of typhoid fever, are, in like manner, very peculiar. It is difficult to describe them in words, but they bear some resemblance to those obtained from the phosphate of ammonia. This kind of crystals is never seen in a state of health; while, on the other hand, the author has never seen them absent in typhoid fever, during many years that he has turned his attention to this subject. Typhoid fever, however, is not the only disease in which they exist; similar crystals are found in cases of pneumonia and acute rheumatism.—*Provincial Med. and Surg. Journal*, June 19, 1841.

